# Real-World Effectiveness and Safety of Anti-CGRP Monoclonal Antibodies in the Prevention of High-Frequency Episodic and Chronic Migraine in Romania—A Multicenter Retrospective Observational Study

**DOI:** 10.3390/biomedicines14092125

**Published:** 2026-09-20

**Authors:** Alin Ciubotaru, Albert Vamanu, Cristina Grosu, Victor Constantinescu, Emilian Bogdan Ignat, Laura-Elena Cucu, Daniel Alexa, Alexandra Mastaleru, Bogdan Ionut Pana, Florina Antochi, Adina Maria Roceanu

**Affiliations:** 1 Grigore T. Popa University of Medicine and Pharmacy, 700115 Iasi, Romania; alinciubotaru94@yahoo.com (A.C.); victor.constantinescu@umfiasi.ro (V.C.); emilian.ignat@umfiasi.ro (E.B.I.); daniel.alexa@umfiasi.ro (D.A.); alexandra.mastaleru@umfiasi.ro (A.M.); 2 Basic and Clinical Neuroscience Department, Institute of Psychiatry, Psychology and Neuroscience, King’s College London, London SE5 8AF, UK; albert.vamanu@kcl.ac.uk; 3 Emerald Hospital Bucharest, 020021 Bucharest, Romania; pana.bogdan@yahoo.com; 4 Neurology Department, University Emergency Hospital Bucharest, 020021 Bucharest, Romania; flrant@yahoo.com (F.A.); amr2012mar@gmail.com (A.M.R.); 5 Headache Group of Romanian Society of Neurology, 020021 Bucharest, Romania

**Keywords:** migraine, anti-CGRP monoclonal antibodies, galcanezumab, erenumab, fremanezumab, eptinezumab, real-world evidence, Romania, high-frequency episodic migraine, chronic migraine, medication overuse, treatment response

## Abstract

**Background:** Real-world evidence regarding the effectiveness and safety of anti-calcitonin gene-related peptide (CGRP) monoclonal antibodies in Eastern European populations remains limited. We aimed to evaluate treatment outcomes and safety in Romanian patients with high-frequency episodic migraine (HFEM) and chronic migraine (CM). **Methods:** We conducted a multicenter retrospective observational cohort study including 113 adults with HFEM (*n* = 38) or CM (*n* = 75) who initiated treatment with galcanezumab, erenumab, fremanezumab, or eptinezumab at specialized headache centers in Romania. Monthly migraine days (MMDs) in HFEM and monthly headache days (MHDs) in CM were assessed at the baseline and during follow-up. Treatment outcomes, discontinuation, persistence, and adverse events were evaluated during routine clinical follow-up. **Results:** At month 6, headache frequency was substantially reduced in both groups, with median MMDs decreasing from 10 to 2 in patients with HFEM and median MHDs decreasing from 25 to 6 in patients with CM. A ≥50% reduction in headache frequency was achieved by 78.9% of patients with HFEM and 64.0% of those with CM. Among the patients with CM, 70.7% met the predefined criterion for conversion to episodic migraine at month 6. During follow-up, 22 patients discontinued treatment. Adverse events were reported in 28 patients (24.8%), were predominantly non-serious, and resulted in treatment discontinuation in 3 patients (2.7%). No serious adverse events were documented. Treatment persistence at 12 months was 82.3%. **Conclusions:** In this Romanian real-world cohort, anti-CGRP monoclonal antibody treatment was associated with substantial reductions in migraine/headache frequency in patients with HFEM and CM. The treatment was generally well-tolerated, with predominantly non-serious adverse events and a low rate of discontinuation due to adverse events. These findings provide real-world evidence from Romania and support further prospective evaluation of the long-term effectiveness and safety of anti-CGRP monoclonal antibodies.

## 1. Introduction

Migraine is a common and severely disabling neurological disorder, affecting approximately 15% of the global population and ranking among the leading causes of disability worldwide [1]. Chronic migraine (CM) and high-frequency episodic migraine (HFEM) are associated with substantial disability, reduced quality of life, and considerable socioeconomic burden [2]. Patients with CM and HFEM frequently require preventive treatment; however, conventional oral preventive therapies may provide suboptimal efficacy and are often limited by tolerability and adherence problems [3].

The preventive treatment of migraine has advanced substantially with the introduction of monoclonal antibodies targeting the calcitonin gene-related peptide (CGRP) pathway or its receptor. CGRP is a potent vasodilatory neuropeptide released from trigeminal terminals during migraine attacks and plays an important role in the trigeminovascular mechanisms underlying migraine pain [4]. Randomized controlled trials have demonstrated the efficacy and safety of erenumab, galcanezumab, fremanezumab, and eptinezumab in the prevention of episodic and chronic migraine [5,6,7,8,9,10,11,12,13]. Galcanezumab has demonstrated efficacy in episodic and chronic migraine in the EVOLVE-1, EVOLVE-2, and REGAIN trials [7,8,9], including in patients with multiple previous preventive treatment failures in the CONQUER study [14]. Fremanezumab, erenumab, and eptinezumab have likewise demonstrated efficacy across relevant episodic and chronic migraine populations [5,6,10,11,12,13].

However, the selected populations and controlled conditions of randomized clinical trials may not fully reflect the complexity of patients encountered in routine clinical practice. Strict eligibility criteria may limit the inclusion of patients with comorbidities, previous treatment failures, medication overuse, or other characteristics commonly encountered in specialized headache care. Therefore, observational real-world studies are important for assessing the effectiveness, safety, treatment persistence, and clinical outcomes of anti-CGRP monoclonal antibodies under routine clinical conditions [15,16].

Real-world studies from Europe have provided evidence supporting the effectiveness of anti-CGRP monoclonal antibodies in routine care. The GARLIT study, conducted in Italy in 163 patients treated with galcanezumab, demonstrated significant reductions in migraine/headache days after 6 months of treatment, with ≥50% responder rates of 76.5% in patients with HFEM and 63.5% in those with CM [15]. Similarly, the EARLY study reported favorable real-world outcomes with erenumab, including a ≥50% responder rate of 74.6% at 6 months [16]. Other real-world studies have also reported clinically meaningful outcomes with fremanezumab and eptinezumab [17,18].

Despite these data, real-world evidence regarding the effectiveness and safety of anti-CGRP monoclonal antibodies in Romania remains limited. Anti-CGRP monoclonal antibodies are available for the preventive treatment of HFEM and CM according to eligibility criteria established by national authorities, but most published real-world evidence originates from Western European countries and the United States [15,16,17,18]. Differences in healthcare access, reimbursement frameworks, patient characteristics, and prescribing practices may limit the direct transferability of findings from these settings to the Romanian healthcare system. Locally generated evidence is therefore needed to characterize treatment outcomes and safety in Romanian patients receiving anti-CGRP monoclonal antibodies in routine clinical practice.

The present study addresses this gap by providing multicenter real-world data from Romania on the effectiveness and safety of four anti-CGRP monoclonal antibodies—galcanezumab, erenumab, fremanezumab, and eptinezumab—in patients with both HFEM and CM. The inclusion of different anti-CGRP agents and longitudinal follow-up with primary efficacy assessments through 12 months allows for the evaluation of headache-frequency outcomes and treatment persistence under routine clinical conditions. By including patients with different migraine phenotypes and medication-overuse status, the study aims to provide evidence that reflects the clinical complexity of patients encountered in specialized headache care.

Therefore, this multicenter retrospective observational study aimed to evaluate the real-world effectiveness and safety of anti-CGRP monoclonal antibodies in Romanian patients with HFEM and CM. The primary outcome was the change in monthly migraine days (MMDs) in patients with HFEM and monthly headache days (MHDs) in patients with CM from the baseline to 3, 6, and 12 months of treatment.

## 2. Materials and Methods

### 2.1. Study Design

This was a multicenter, retrospective, observational cohort study conducted in Romania to evaluate the real-world effectiveness, safety, treatment persistence, and clinical outcomes associated with anti-calcitonin gene-related peptide (CGRP) monoclonal antibody therapy in adults with high-frequency episodic migraine (HFEM) and chronic migraine (CM). The study was based on a retrospective analysis of clinical data routinely recorded during the management of eligible patients at participating specialized headache centers. The analyzed information was obtained from electronic and paper-based medical records and included demographic characteristics, migraine phenotype and history, previous preventive treatment exposure, headache frequency, acute medication use, patient-reported clinical outcomes, adverse events, treatment discontinuation, and longitudinal treatment response. The underlying clinical data were collected during routine clinical follow-up and subsequently analyzed retrospectively; no additional diagnostic procedures, therapeutic interventions, or treatment allocation were performed as part of the research protocol.

Treatment selection and subsequent clinical management remained the responsibility of the treating physician and were based on individual clinical characteristics, previous preventive treatment history, applicable prescribing information, and national eligibility and reimbursement criteria. Accordingly, the study did not involve randomization or protocol-driven treatment allocation. The study was conducted in accordance with the principles of the Declaration of Helsinki and applicable national regulations. Ethical approval was obtained from the Institutional Ethics Committees of the participating centers before the extraction and analysis of clinical data.

### 2.2. Study Setting and Participating Centers

The study was conducted across specialized headache-care centers in Romania, including the Headache Center, Neurology Clinic, “Grigore T. Popa” University of Medicine and Pharmacy, Iași; the Headache Center, University Emergency Hospital Bucharest, Bucharest; and Emerald Medical Centers, Romania as well as the participating clinical sites contributing eligible patients to the present cohort. These centers provide specialized neurological and headache care and routinely manage patients with episodic and chronic migraine, including patients receiving anti-CGRP monoclonal antibody therapy. Patients were evaluated and followed by physicians experienced in headache management, and treatment decisions were made according to routine clinical practice, applicable international recommendations, prescribing information, and national reimbursement and eligibility criteria. The multicenter design allowed for the analysis of routinely collected clinical data from different specialized headache-care settings and was intended to characterize the effectiveness and safety of anti-CGRP monoclonal antibodies under real-world conditions in Romania.

### 2.3. Study Population

A total of 200 patients were initially assessed for eligibility. After application of the predefined inclusion and exclusion criteria, 113 patients were included in the final study cohort, while 87 patients were excluded. The study population consisted of adults with HFEM or CM who initiated treatment with an anti-CGRP monoclonal antibody during the predefined study period and fulfilled the eligibility criteria. The documented reasons for exclusion included insufficient clinical data, treatment discontinuation, non-adherence to treatment, and pregnancy, with two patients excluded because they became pregnant.

#### 2.3.1. Inclusion Criteria

Patients were eligible for inclusion if they met all predefined criteria: (1) age ≥18 years at treatment initiation; (2) diagnosis of HFEM, defined as 8–14 migraine days per month, or CM, defined as ≥15 headache days per month, of which at least 8 had migraine features, according to the International Classification of Headache Disorders, 3rd edition (ICHD-3); (3) initiation of treatment with an anti-CGRP monoclonal antibody, including galcanezumab, erenumab, fremanezumab, or eptinezumab, according to the applicable national eligibility criteria; (4) previous failure of preventive migraine treatment according to the eligibility criteria applied in routine clinical practice; (5) availability of baseline clinical data and at least one eligible follow-up assessment; (6) availability of sufficient clinical information to permit evaluation of the predefined study outcomes; and (7) availability of consent for the use of clinical data for research purposes, in accordance with the procedures approved by the participating institutions.

#### 2.3.2. Exclusion Criteria

Patients were excluded if they met any predefined exclusion criterion, including: (1) previous participation in a clinical trial involving an anti-CGRP monoclonal antibody; (2) pregnancy or breastfeeding at treatment initiation; (3) known hypersensitivity to the active substance or any of the excipients; (4) contraindications to treatment with the respective anti-CGRP monoclonal antibody; (5) absence of an adequate baseline clinical assessment; (6) insufficient follow-up information for the predefined analyses; or (7) absence of the required authorization or consent for research use of clinical data, where applicable according to institutional procedures.

### 2.4. Anti-CGRP Monoclonal Antibody Treatment

Patients received one of four anti-CGRP monoclonal antibodies available in routine clinical practice during the study period. Treatment regimens were as follows: (1) galcanezumab (Emgality^®^), administered as a loading dose of 240 mg subcutaneously as two 120 mg injections, followed by 120 mg subcutaneously once monthly [10]; (2) erenumab (Aimovig^®^), administered at a dose of 70 mg or 140 mg subcutaneously once monthly [7]; (3) fremanezumab (Ajovy^®^), administered at a dose of 225 mg subcutaneously once monthly or 675 mg subcutaneously once every 3 months [12]; and (4) eptinezumab (Vyepti^®^), administered at a dose of 100 mg or 300 mg intravenously once every 3 months [13]. Treatment was administered according to the prescribing information for the respective products, applicable clinical recommendations, and local clinical protocols. The choice of monoclonal antibody, dose, continuation or discontinuation of treatment, and subsequent therapeutic decisions were made by the treating physician; no treatment assignment was performed by the study investigators.

### 2.5. Data Collection

Clinical data were retrospectively extracted from electronic and paper-based medical records of eligible patients treated at the participating headache centers. Data extraction was performed by physicians involved in the clinical management of the patients using a standardized data collection framework. The collected variables were selected according to the predefined study objectives and included demographic characteristics; migraine history and phenotype; headache frequency; acute medication use; pain intensity; disability and headache-impact measures; previous preventive treatments; medication overuse; comorbidities; concomitant medication; treatment exposure; clinical response; adverse events; and treatment discontinuation.

At treatment initiation, the recorded variables included age and sex; age at migraine onset and disease duration; migraine phenotype (HFEM or CM); presence of aura; MMDs in patients with HFEM; MHDs in patients with CM; monthly acute medication use; pain intensity assessed using the Numerical Rating Scale (NRS), ranging from 0 to 10; HIT-6 score; MIDAS score; medication overuse according to ICHD-3 criteria; previous preventive treatments, including type and number of previous treatment failures; acute treatments, including type and frequency of use; comorbidities; and concomitant medication.

Clinical information was assessed longitudinally at predefined follow-up time points of months 1, 3, 6, and 12 after treatment initiation, according to the availability of clinical documentation. Variables assessed during follow-up included MMD/MHD, monthly acute medication use, NRS pain intensity, HIT-6 score, MIDAS score, adverse events, treatment discontinuation and its documented reason, conversion from CM to episodic migraine, and discontinuation of medication overuse. HIT-6 and MIDAS assessments were available at months 3, 6, and 12, whereas MMD/MHD, acute medication use, and NRS were assessed at months 1, 3, 6, and 12. Patients were included in a given time-point analysis when the corresponding follow-up information was available, and missing individual observations were handled according to the predefined last observation carried forward (LOCF) approach. Adverse events were recorded irrespective of whether a causal relationship with the anti-CGRP monoclonal antibody could be established.

#### Clinical Assessment Instruments

Clinical assessments were performed using instruments routinely employed in headache practice. The headache diary was used to record headache occurrence, headache characteristics, intensity, and acute medication use. Pain severity was assessed using the Numerical Rating Scale (NRS), ranging from 0 to 10. The Headache Impact Test (HIT-6) is a six-item patient-reported instrument assessing the impact of headache on daily functioning and quality of life, with scores ranging from 36 to 78 and higher scores indicating greater headache-related impact. The Migraine Disability Assessment (MIDAS) questionnaire was used to assess migraine-related disability, with higher scores indicating greater disability.

### 2.6. Study Outcomes and Variables

#### 2.6.1. Primary Outcome

The primary efficacy outcome was the change in headache frequency from the baseline to follow-up, expressed as the change in monthly migraine days (MMDs) among patients with HFEM and the change in monthly headache days (MHDs) among patients with CM. Changes were evaluated longitudinally at months 3, 6, and 12.

#### 2.6.2. Secondary Outcomes

Secondary outcomes included: (1) change in monthly acute medication use from the baseline to months 3, 6, and 12; (2) change in NRS pain intensity from the baseline to months 3, 6, and 12; (3) change in HIT-6 and MIDAS scores from the baseline to months 3, 6, and 12; (4) proportions of patients achieving ≥50%, ≥75%, and 100% reductions in MMD/MHD at months 3, 6, and 12; (5) conversion from CM to episodic migraine at months 3, 6, and 12; (6) discontinuation of medication overuse at months 3, 6, and 12; (7) incidence and type of reported adverse events; and (8) treatment discontinuation and documented reasons for discontinuation.

#### 2.6.3. Variables Considered in Exploratory Response Analysis

Baseline variables considered in the exploratory analysis of treatment response included age, sex, migraine phenotype, presence of aura, medication overuse, number of previous preventive treatment failures, disease duration, body mass index, baseline MMD/MHD, baseline MIDAS score, baseline HIT-6 score, and relevant comorbidities.

### 2.7. Study Period and Follow-Up

**Figure 1:**
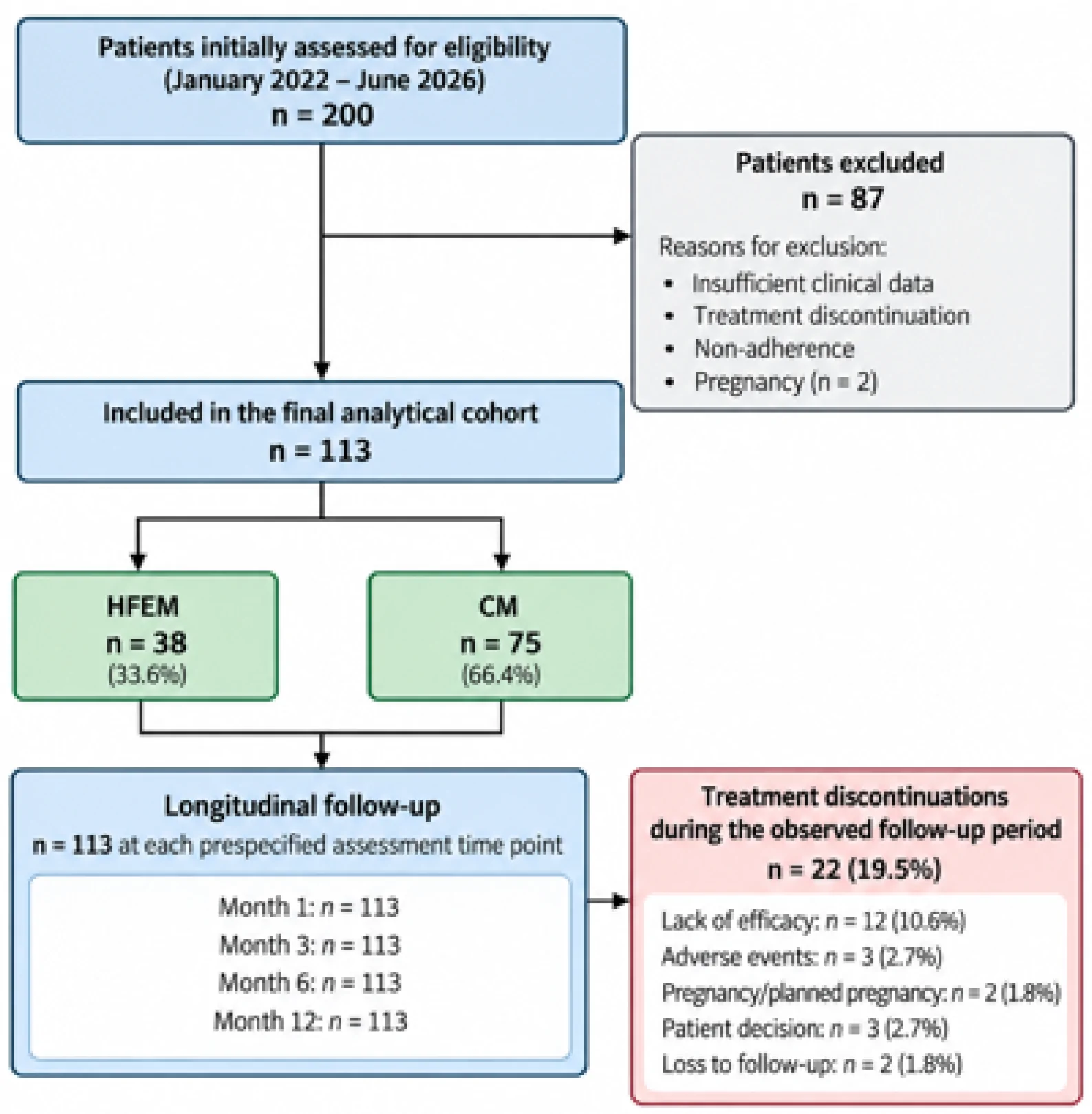
STROBE participant flow diagram. The diagram summarizes patient eligibility assessment, exclusions, inclusion in the final analytical cohort, longitudinal follow-up at months 1, 3, 6, and 12, and treatment discontinuations during the observed follow-up period. HFEM, high-frequency episodic migraine; CM, chronic migraine.

The study included patients who initiated anti-CGRP monoclonal antibody treatment during the predefined study period. The retrospective observational design means that this period represents treatment initiation and patient eligibility rather than prospective recruitment. The predefined follow-up assessments were scheduled at months 1, 3, 6, and 12, while additional information from routine clinical follow-up was available for some patients beyond 12 months. The principal efficacy analyses were performed at the baseline and at months 3, 6, and 12, with information collected at month 1 used for corresponding early-response analyses. Patients with longer available follow-up were considered for analyses of treatment persistence and discontinuation according to the availability of longitudinal clinical documentation. Participant selection, exclusions, longitudinal follow-up, and treatment discontinuations are summarized in Figure 1.

### 2.8. Statistical Analysis

All statistical analyses were performed using IBM SPSS Statistics version 27.0 (IBM Corp., Armonk, NY, USA). All statistical tests were two-sided, and a *p*-value < 0.05 was considered statistically significant.

#### 2.8.1. Analysis Population and Missing Data

The efficacy analysis included eligible patients who had received at least one dose of an anti-CGRP monoclonal antibody, had baseline information, and had at least one post-baseline clinical assessment. Patients who discontinued treatment after at least one post-baseline assessment remained eligible for longitudinal analyses up to the point of discontinuation, whereas patients who discontinued treatment before any post-baseline assessment could not contribute to efficacy analyses because no post-treatment outcome was available. For longitudinal analyses, missing observations were handled using the last observation carried forward (LOCF) approach, as specified in the original analytical plan. This approach was consistent with the methodology reported in comparable real-world evidence, including the GARLIT study [15], although the potential for attrition and imputation-related bias was considered when interpreting the findings. For safety analyses, all patients who received at least one dose of treatment were included, irrespective of the availability of complete efficacy follow-up.

#### 2.8.2. Descriptive Statistics

Continuous variables were assessed for distributional characteristics using the Kolmogorov–Smirnov test. Normally distributed continuous variables were summarized as mean ± standard deviation (SD), whereas non-normally distributed variables were presented as median and interquartile range (IQR). Categorical variables were summarized as absolute frequencies and percentages. Baseline demographic and clinical characteristics were presented for the overall cohort, and where appropriate, according to migraine phenotype.

#### 2.8.3. Longitudinal Analysis

Changes in MMD/MHD, monthly acute medication use, NRS, HIT-6, and MIDAS scores over time were assessed using non-parametric methods when distributional assumptions for parametric testing were not met. For repeated measurements across multiple time points, the Friedman test was used for the overall comparison; when a statistically significant overall difference was identified, pairwise comparisons were performed using the Wilcoxon signed-rank test with Bonferroni correction for multiple comparisons. The primary efficacy comparison of MMD/MHD between the baseline and month 6 was performed using the Wilcoxon signed-rank test for paired observations. The statistical methodology was consistent with the non-parametric analytical approach described in the original study protocol and supported by the cited statistical methodology [19]. The use of LOCF for missing longitudinal observations was taken into account when interpreting repeated-measures and paired analyses because of the potential for imputation-related bias.

#### 2.8.4. Responder Rate Analysis

Responder rates were calculated at months 1, 3, 6, and 12. Patients were categorized according to the percentage reduction in MMD/MHD relative to the baseline as follows: (1) ≥50% responders, defined as a ≥50% reduction in MMD/MHD; (2) ≥75% responders, defined as a ≥75% reduction in MMD/MHD; and (3) 100% responders, defined as complete reduction to zero MMD/MHD. Responder rates were reported as percentages with corresponding 95% confidence intervals (CIs), calculated using the Wilson score method, consistent with the approach reported in the GARLIT study [15].

#### 2.8.5. Subgroup Analyses

Patients were classified as responders or non-responders according to whether they achieved a ≥50% reduction in MMD/MHD at month 6. Baseline demographic and clinical characteristics were compared between responder and non-responder groups using the independent-samples *t*-test for continuous variables with an approximately normal distribution, the Mann–Whitney U test for non-normally distributed continuous variables, and the chi-square test or Fisher’s exact test for categorical variables, as appropriate. Among the patients with CM, conversion to episodic migraine was defined as a reduction to <15 headache days per month and was assessed at months 3, 6, and 12 [15]. Among patients fulfilling criteria for medication overuse at the baseline, discontinuation of medication overuse was defined as no longer fulfilling the applicable ICHD-3 criteria for medication overuse and was assessed at months 3, 6, and 12.

#### 2.8.6. Exploratory Predictive Factors Analysis

An exploratory binary logistic regression analysis was performed to investigate baseline factors associated with achieving a ≥50% reduction in MMD/MHD at month 6. Variables showing an association with treatment response at *p* < 0.10 in univariate analysis were entered into the multivariable model using the forced-entry method. Candidate variables included age, sex, migraine phenotype, presence of aura, medication overuse, number of previous preventive treatment failures, disease duration, body mass index, baseline MMD/MHD, baseline MIDAS score, and baseline HIT-6 score. Results were expressed as adjusted odds ratios (ORs) with 95% CIs and *p*-values. Goodness-of-fit of the final logistic regression model was assessed using the Hosmer–Lemeshow test. For this analysis, achieving a ≥50% reduction in MMD/MHD at month 6 was defined as the binary outcome; among the 113 patients in the analytical cohort, 78 met the ≥50% response criterion and 35 did not, and the final multivariable model included five predictor variables, corresponding to an events-per-variable ratio of 15.6.

#### 2.8.7. Treatment Discontinuation and Persistence

Treatment discontinuation was assessed according to the clinical records. The proportion of patients who discontinued treatment and the documented reasons for discontinuation were recorded, including lack of efficacy, adverse events, patient decision, pregnancy, and other clinically documented reasons. For patients who discontinued treatment, the time from treatment initiation to discontinuation was recorded in months. Treatment persistence was analyzed using the Kaplan–Meier method, with treatment discontinuation defined as the event of interest; patients who remained on treatment at the end of the available follow-up period were censored at their last documented follow-up date. Kaplan–Meier estimates of treatment persistence were calculated at 6, 12, and 18 months, and the corresponding survival probabilities represented the estimated proportion of patients remaining on treatment at each time point. The median time to treatment discontinuation was also estimated from the Kaplan–Meier analysis.

#### 2.8.8. Sample Size Considerations

Based on published real-world evidence and preliminary data available from the participating centers, the expected reduction in MMD/MHD after 6 months of treatment was estimated at approximately 8–10 days, with an assumed standard deviation of approximately 6 days. For a two-sided significance level of α = 0.05 and 80% statistical power, the estimated minimum sample size was 50 patients. To allow for subgroup analyses and potential loss to follow-up, the study aimed to include at least 100 patients. Based on the available clinical population across the participating centers, this target was considered achievable.

#### 2.8.9. Statistical Software

All statistical analyses were performed using IBM SPSS Statistics version 27.0 (IBM Corp., Armonk, NY, USA). All statistical tests were two-sided, and statistical significance was defined as *p* < 0.05.

### 2.9. Ethical Considerations

#### 2.9.1. Ethical Approval and Regulatory Framework

The study was conducted in accordance with the principles of the Declaration of Helsinki (2013 revision) and applicable national regulations governing clinical research and the use of patient data. The study protocol was submitted to and approved by the Institutional Ethics Committee of each participating center before the extraction and analysis of clinical data.

#### 2.9.2. Informed Consent and Data Protection

Because the study involved the retrospective analysis of routinely collected clinical data, the use of patient information for research purposes was performed in accordance with the consent procedures and data-protection requirements approved by the participating institutions. Where informed consent for the research use of clinical data was obtained at treatment initiation, this consent included the use of appropriately anonymized clinical information for scientific analysis and publication. All data used for analysis were handled in a manner intended to protect patient confidentiality, and personally identifiable information was not included in the analytical dataset or manuscript.

## 3. Results

### 3.1. Participant Characteristics

A total of 200 patients were initially assessed for eligibility. Following application of the predefined inclusion and exclusion criteria, 113 patients were included in the final analytical cohort, while 87 patients were excluded. The documented reasons for exclusion included insufficient clinical data, treatment discontinuation, non-adherence, and pregnancy, with 2 patients excluded because of pregnancy. The final cohort consisted of 38 patients with high-frequency episodic migraine (HFEM) and 75 patients with chronic migraine (CM). The availability of longitudinal outcome data varied according to the timing and completeness of routine clinical follow-up. Patients who had at least one post-baseline assessment contributed to the efficacy analyses, while patients who discontinued treatment remained eligible for analyses up to the point of discontinuation.

The participant flow is presented in Figure 1.

Of these, 108 patients (95.6%) were female and 5 (4.4%) were male. The mean age at treatment initiation was 42.6 ± 10.8 years (range, 18–75 years), while the mean duration of migraine disease was 15.3 ± 9.7 years (range, 2–47 years).

Among the 113 participants, 38 patients (33.6%) had HFEM, defined as 8–14 migraine days per month, whereas 75 patients (66.4%) had CM, defined as ≥15 headache days per month, with at least 8 days presenting migraine features according to ICHD-3 criteria. Aura was documented in 17 patients (15.0%).

Medication overuse was present at the baseline in 67 patients (59.3%). The prevalence of medication overuse was significantly higher among patients with CM than among those with HFEM (57/75 [76.0%] vs. 10/38 [26.3%], *p* < 0.001). This finding is consistent with the recognized association between medication overuse and migraine chronification [3].

The mean number of previous preventive treatment failures was 2.8 ± 1.4 (range, 1–7). Patients with CM had a significantly higher number of previous preventive treatment failures than patients with HFEM (3.0 ± 1.5 vs. 2.4 ± 1.2, *p* = 0.026).

Among the previously used preventive therapies, the most frequently documented treatment failures were beta-blockers (*n* = 89, 78.8%), topiramate (*n* = 76, 67.3%), amitriptyline (*n* = 68, 60.2%), and onabotulinumtoxinA (*n* = 32, 28.3%). These therapies represent commonly used preventive treatment options in migraine management [4,5].

Baseline demographic and clinical characteristics according to migraine phenotype are summarized in Table 1.

Patients with CM had a significantly longer disease duration than those with HFEM (16.6 ± 10.1 vs. 12.8 ± 8.4 years, *p* = 0.041). No significant between-group differences were observed for age (*p* = 0.124), sex (*p* = 0.762), aura (*p* = 0.695), or body mass index (*p* = 0.183).

In contrast, several measures of baseline disease burden were significantly higher in the CM group. Baseline MMD/MHD was higher among patients with CM than among those with HFEM (median 25 [IQR, 18–30] vs. 10 [IQR, 8–12], *p* < 0.001). Similarly, baseline monthly painkiller intake (MPI) was higher in the CM group (20 [15,16,17,18,20,21,22,23,24,25,26] vs. 10 [6,7,8,9,10,11,12,13,14], *p* < 0.001). Baseline NRS scores were also significantly higher among patients with CM (8 [7,8,9] vs. 7 [6,7,8], *p* = 0.038).

Measures of headache-related impact and disability showed the same pattern. Median baseline HIT-6 scores were 70 (IQR, 66–72) in patients with CM compared with 64 (IQR, 60–68) in patients with HFEM (*p* < 0.001), while median MIDAS scores were 70 (IQR, 56–80) and 30 (IQR, 25–50), respectively (*p* < 0.001).

Overall, these findings indicate that the CM subgroup had a greater baseline burden of headache frequency, acute medication use, pain intensity, headache-related impact, and migraine-related disability than the HFEM subgroup.

Regarding treatment exposure, 89 patients (78.8%) received galcanezumab, 12 (10.6%) received erenumab, 9 (8.0%) received fremanezumab, and 3 (2.6%) received eptinezumab.

Thus, galcanezumab represented the predominant treatment in the study cohort, accounting for approximately four-fifths of all treatment exposures, whereas the other three monoclonal antibodies were administered to smaller subgroups.

Given the unequal distribution of treatment exposure across the four agents, particularly the small number of patients receiving eptinezumab, the treatment groups were not considered sufficiently balanced to support robust comparative effectiveness analyses between individual monoclonal antibodies.

### 3.2. Primary Outcome: Change in Monthly Migraine/Headache Days

Among the 38 patients with high-frequency episodic migraine (HFEM), the median baseline number of monthly migraine days (MMDs) was 10 (IQR, 8–12). Following the initiation of anti-CGRP monoclonal antibody treatment, a significant reduction in MMDs was observed throughout the follow-up period, with statistically significant differences at each assessment compared with the baseline (*p* < 0.001 for all comparisons).

An early reduction in migraine frequency was already evident at the first follow-up assessment. At month 1, median MMDs decreased from 10 (IQR, 8–12) at the baseline to 4 (IQR, 2–6), corresponding to an absolute reduction of 6 migraine days based on the group medians. At month 3, the median MMDs further decreased to 3 (IQR, 1–5). At month 6, median MMDs reached 2 (IQR, 1–4), and this reduction was maintained at month 12, when the median MMDs remained 2 (IQR, 0–4).

The early reduction in migraine frequency observed during the first month of treatment is consistent with the relatively rapid onset of clinical benefit reported for galcanezumab and other anti-CGRP monoclonal antibodies [6,7]. The persistence of the reduction through 12 months suggests the sustained effectiveness of anti-CGRP treatment in this HFEM cohort.

The mean reduction in MMDs from the baseline to month 6 was 7.2 ± 3.8 days (95% CI, 5.9–8.5 days). Relative to the mean baseline MMDs used for this analysis, this corresponded to a mean percentage reduction of 72.0%. This magnitude of reduction exceeds the ≥50% response threshold commonly used to define a clinically meaningful treatment response in migraine preventive studies [8].

At month 12, the median MMDs remained at 2 days (IQR, 0–4), indicating that the reduction in migraine frequency observed during the first 6 months was maintained during longer-term follow-up. The persistence of this response is clinically relevant given the recurrent nature of migraine and the need for sustained preventive treatment.

Among the 75 patients with chronic migraine (CM), the median baseline number of monthly headache days (MHDs) was 25 (IQR, 18–30). Following the initiation of anti-CGRP monoclonal antibody treatment, a significant reduction in MHDs was observed throughout the follow-up period, with statistically significant differences at each assessment compared with the baseline (*p* < 0.001 for all comparisons).

An early reduction in headache frequency was already evident at the first follow-up assessment. At month 1, the median MHDs decreased from 25 (IQR, 18–30) at the baseline to 10 (IQR, 6–15), corresponding to an absolute reduction of 15 headache days based on the group medians. At month 3, the median MHDs further decreased to 8 (IQR, 4–12). At month 6, median MHDs reached 6 (IQR, 3–10), and the reduction was maintained at month 12, when the median MHDs was 5 (IQR, 2–8).

The mean reduction in MHDs from the baseline to month 6 was 16.2 ± 7.4 days (95% CI, 14.5–17.9 days). Relative to the mean baseline value used for this analysis, this corresponded to a mean percentage reduction of 64.8%. The magnitude of this reduction indicates a substantial decrease in headache frequency during treatment.

At month 12, the median MHDs was 5 days per month (IQR, 2–8), which is below the diagnostic threshold for chronic migraine of ≥15 headache days per month. However, the median value alone does not provide the proportion of individual patients who converted from CM to episodic migraine. Therefore, the conversion rate was assessed separately according to the predefined study criterion and is reported in the corresponding analysis of migraine phenotype conversion.

The larger reduction observed in the present cohort may reflect, at least in part, differences in baseline headache frequency and other clinical characteristics, the real-world observational setting, and the longer assessment interval used for the principal comparison. 

### 3.3. Secondary Outcomes

The reduction in monthly analgesic intake (MPI) paralleled the reduction in migraine and headache frequency observed during anti-CGRP monoclonal antibody treatment. This pattern was observed in both the HFEM and CM groups and was accompanied by a significant reduction in acute medication use during follow-up.

Among patients with HFEM, the median monthly analgesic intake decreased from 10 (IQR, 6–14) at the baseline to 3 (IQR, 1–5) at month 6 (*p* < 0.001), corresponding to an absolute reduction of 7 days based on the group medians. This represents a 70.0% reduction when calculated from the reported median values.

The reduction was maintained at month 12, when the median MPI was 2 days (IQR, 1–4).

Among patients with CM, the median monthly analgesic intake decreased from 20 (IQR, 15–25) at the baseline to 6 (IQR, 3–10) at month 6 (*p* < 0.001), corresponding to an absolute reduction of 14 days based on the group medians and a 70.0% reduction calculated from the reported median values. The reduction was maintained at month 12, when the median MPI was 4 days (IQR, 2–8).

The reduction in acute medication use was particularly relevant in the CM subgroup, in which medication overuse was present in 76.0% of patients at the baseline. The observed decrease in monthly analgesic intake is therefore consistent with the reduction in medication overuse documented during follow-up and may be clinically relevant to the management of medication-overuse headache [3].

Overall, the parallel decrease in headache frequency and monthly analgesic intake suggests a reduction in the need for acute medication during preventive treatment. Parallel decrease in headache frequency and monthly analgesic intake suggests a reduction in the need for acute medication during preventive treatment. Pain intensity was assessed using the 0–10 Numerical Rating Scale (NRS). A significant reduction in NRS scores was observed after treatment with anti-CGRP monoclonal antibodies in both migraine subgroups.

Among the patients with HFEM, the median NRS score decreased from 7 (IQR, 6–8) at the baseline to 5 (IQR, 4–6) at month 6 (*p* < 0.001), corresponding to an absolute reduction of 2 points based on the group medians. This represents a 28.6% reduction calculated from the reported median values. Among the patients with CM, the median NRS score decreased from 8 (IQR, 7–9) at the baseline to 5 (IQR, 4–6) at month 6 (*p* < 0.001), corresponding to an absolute reduction of 3 points based on the group medians and a 37.5% reduction calculated from the reported median values.

The magnitude of the observed reduction was clinically relevant in both subgroups. In particular, the reduction of at least 2 points observed in the HFEM group and 3 points in the CM group met the predefined threshold considered indicative of a clinically meaningful change in pain intensity [11].

The greater absolute reduction observed in the CM group occurred in the context of a higher baseline median NRS score (8 vs. 7 points in HFEM). A significant improvement in headache-related impact and migraine-related disability was observed in both the HFEM and CM groups following treatment with anti-CGRP monoclonal antibodies. The changes observed across the secondary patient-reported outcomes at month 6 are summarized in Table 2.

Among the patients with HFEM, the median HIT-6 score decreased from 64 (IQR, 60–68) at the baseline to 52 (IQR, 48–56) at month 6 (*p* < 0.001), corresponding to an absolute reduction of 12 points based on the group medians. This magnitude of change exceeded the 8-point threshold considered the minimal clinically important difference (MCID) for HIT-6 [12]. In the CM group, the median HIT-6 score decreased from 70 (IQR, 66–72) at the baseline to 55 (IQR, 50–60) at month 6 (*p* < 0.001), corresponding to an absolute reduction of 15 points based on the group medians. This change also exceeded the reported MCID threshold [12].

Migraine-related disability, assessed using the MIDAS questionnaire, showed a significant improvement in both groups.

In the HFEM group, the median MIDAS score decreased from 30 (IQR, 25–50) at the baseline to 8 (IQR, 3–15) at month 6 (*p* < 0.001), corresponding to an absolute reduction of 22 points based on the group medians and a 73.3% reduction calculated from the reported median values. In the CM group, the median MIDAS score decreased from 70 (IQR, 56–80) at the baseline to 20 (IQR, 10–35) at month 6 (*p* < 0.001), corresponding to an absolute reduction of 50 points based on the group medians and a 71.4% reduction calculated from the reported median values.

The median MIDAS score in the HFEM group decreased from a value corresponding to Grade IV disability (>21) at the baseline to a value corresponding to Grade II disability (6–10) at month 6. In the CM group, the median score decreased from Grade IV disability at the baseline to a value corresponding to Grade III disability (11–20) at month 6.

The magnitude and direction of the changes observed across MPI, NRS, HIT-6, and MIDAS were consistent, with all assessed secondary outcomes showing a statistically significant improvement at month 6 compared with the baseline (*p* < 0.001 for all comparisons; Table 2). The median changes from the baseline were −7 and −14 days for MPI, −2 and −3 points for NRS, −12 and −15 points for HIT-6, and −22 and −50 points for MIDAS in the HFEM and CM groups, respectively (Table 2).

Overall, the improvement in HIT-6 and MIDAS scores complements the reductions observed in headache frequency, acute medication use, and pain intensity, indicating a substantial reduction in the patient-reported burden of migraine following anti-CGRP monoclonal antibody treatment [13].

### 3.4. Responder Rates

The proportion of patients achieving a ≥50% reduction in MMD/MHD increased during the first 3–6 months of treatment in both migraine subgroups, with a sustained response observed at month 12.

Responder analyses included 113 patients at each prespecified assessment time point (38 with HFEM and 75 with CM at months 1, 3, 6, and 12). The responder rate increased to 27/38 (71.1%; 95% CI, 55.0–83.5%) at month 3 and reached 30/38 (78.9%; 95% CI, 63.5–89.2%) at month 6. At month 12, 28/38 patients (73.7%; 95% CI, 57.9–85.3%) continued to meet the ≥50% response criterion.

Among the patients with CM, 40/75 patients (53.3%; 95% CI, 42.0–64.2%) achieved a ≥50% reduction in MHDs at month 1. The responder rate increased to 47/75 (62.7%; 95% CI, 51.3–72.8%) at month 3 and reached 48/75 (64.0%; 95% CI, 52.6–74.1%) at month 6. At month 12, 45/75 patients (60.0%; 95% CI, 48.5–70.5%) remained responders.

Thus, the highest observed ≥50% responder rates occurred at month 6 in both groups, followed by a modest reduction at month 12, while remaining above the corresponding month 1 rates. The responder rates at each assessment time point are summarized in Table 3.

A substantial proportion of patients achieved a ≥75% reduction in migraine or headache frequency during follow-up. In the HFEM group, 13/38 patients (34.2%; 95% CI, 20.6–50.7%) achieved a ≥75% reduction in MMDs at month 6, while 12/38 patients (31.6%; 95% CI, 18.6–47.9%) met this criterion at month 12.

In the CM group, 25/75 patients (33.3%; 95% CI, 23.5–44.6%) achieved a ≥75% reduction in MHDs at month 6, while 22/75 patients (29.3%; 95% CI, 20.0–40.6%) met the criterion at month 12. Therefore, approximately one-third of patients in both migraine subgroups achieved a ≥75% reduction in headache frequency at month 6, with the response maintained in approximately 30% of patients at month 12.

The ≥75% responder rate represents a more stringent treatment-response threshold than the conventional ≥50% criterion and identifies patients experiencing a marked reduction in migraine or headache frequency. However, these responder rates should be interpreted in the context of the baseline disease burden and should not by themselves be equated with normalization of daily functioning or complete clinical remission.

Complete elimination of migraine or headache days at the respective assessment point was observed in a smaller proportion of patients. In the HFEM group, 4/38 patients (10.5%; 95% CI, 3.7–24.6%) achieved a 100% response at month 6, increasing to 5/38 patients (13.2%; 95% CI, 5.2–27.7%) at month 12. In the CM group, 5/75 patients (6.7%; 95% CI, 2.6–15.0%) achieved a 100% response at month 6, while 6/75 patients (8.0%; 95% CI, 3.3–16.5%) met this criterion at month 12.

Overall, the proportion of patients achieving zero migraine/headache days at the respective assessment point ranged from 6.7% to 13.2% across the two migraine subgroups and follow-up assessments.

The 100% response rate represents a stringent endpoint and was observed in a minority of patients. Complete absence of migraine/headache days was maintained in a subset of patients at month 12.

### 3.5. Conversion from Chronic to Episodic Migraine

Conversion from chronic to episodic migraine represented an important clinical outcome in the present cohort. A substantial proportion of patients with baseline chronic migraine moved below the diagnostic frequency threshold during treatment, and this pattern remained broadly stable during subsequent follow-up. Clinically, frequency-based conversion represents a meaningful reduction in headache burden but does not by itself establish long-term disease modification. The conversion rate observed at month 6 indicates that a substantial proportion of patients who initially met the criteria for chronic migraine no longer met the frequency threshold for chronic migraine during treatment. This finding is clinically relevant given the greater disease burden and disability generally associated with chronic compared with episodic migraine [17].

The conversion rate remained relatively stable between months 6 and 12, decreasing only slightly from 70.7% to 69.3%, indicating that the observed conversion was maintained in a substantial proportion of patients during longer-term follow-up. Overall, the findings indicate that conversion from chronic to episodic migraine occurred in approximately two-thirds to seven-tenths of the CM cohort during follow-up, with the highest observed conversion rate at month 6.

### 3.6. Medication Overuse Discontinuation

Among the 67 patients with medication overuse at the baseline, 48 patients (71.6%) no longer fulfilled the predefined criteria for medication overuse at month 3. This proportion increased to 51 patients (76.1%) at month 6 and remained relatively stable at month 12, when 50 patients (74.6%) no longer fulfilled the criteria for medication overuse.

The proportion of patients discontinuing medication overuse at month 6 was therefore 76.1%, indicating that a substantial proportion of patients with baseline medication overuse experienced a reduction in acute medication use sufficient to no longer meet the predefined criteria during treatment. This finding is clinically relevant because medication overuse is associated with increased headache burden and disability and represents an important component of the management of patients with chronic migraine [3].

The reduction in medication overuse occurred in parallel with the substantial reduction in MMD/MHD and monthly analgesic intake observed in the cohort. The proportion of patients no longer fulfilling medication-overuse criteria remained relatively stable between month 6 (76.1%) and month 12 (74.6%). This persistence suggests that the reduction in medication overuse was maintained in most patients who achieved this outcome during the first 6 months of treatment.

Overall, the findings indicate that anti-CGRP monoclonal antibody treatment was associated with substantial reductions in medication overuse in this treatment-experienced cohort. The observed changes are particularly relevant in the context of the high baseline prevalence of medication overuse among patients with CM.

### 3.7. Treatment Discontinuation

Treatment discontinuation occurred in a minority of patients during the observed follow-up period. Lack of efficacy was the most frequently documented reason, while adverse events, pregnancy or planned pregnancy, patient preference, and loss to follow-up accounted for additional discontinuations. This pattern suggests that treatment continuation in routine practice is influenced by both clinical effectiveness and individual treatment circumstances. The relatively limited contribution of adverse events to treatment discontinuation supports an overall favorable observed tolerability profile, while also emphasizing the need for continued monitoring during longer-term preventive treatment.

Lack of efficacy was therefore the most frequently documented reason for treatment discontinuation, accounting for more than half of all discontinuations. Adverse events accounted for a smaller proportion of discontinuations (3/113, 2.7%).

The overall discontinuation rate observed in the present cohort was lower than the discontinuation rates commonly reported with several oral preventive treatments, for which tolerability and lack of efficacy are important determinants of treatment persistence [20]. However, direct comparison with oral preventive therapies should be interpreted cautiously because treatment persistence is influenced by differences in study design, patient populations, treatment duration, clinical monitoring, and criteria for treatment discontinuation.

The median time to treatment discontinuation was 7.5 months (range, 3–18 months) among the patients who discontinued treatment. Kaplan–Meier analysis showed estimated treatment-persistence probabilities of 86.5% at 6 months, 82.3% at 12 months, and 75.2% at 18 months. Patients who had not discontinued treatment by the end of their available follow-up were treated as censored observations in the analysis.

These estimates indicate that most patients remained on anti-CGRP treatment during the observed follow-up period. Published real-world studies have reported variable persistence rates for migraine preventive therapies, including lower persistence with some oral preventive treatments [20,21].

The observed persistence in the present cohort should nevertheless be interpreted in the context of the retrospective study design and the available follow-up data. 

### 3.8. Adverse Events

Adverse events were reported in 28 out of 113 patients (24.8%), including 8 patients with HFEM (21.1%) and 20 with CM (26.7%) (Table 4). The most frequently reported events were local injection-site reactions (7.1%), constipation (5.3%), alopecia (3.5%), and menstrual irregularities (2.7%). Other reported events occurred less frequently. No serious adverse events were documented, and adverse events resulted in treatment discontinuation in 3 patients (2.7%). The distribution of adverse events according to migraine phenotype is presented in Table 4.

### 3.9. Predictive Factors of Treatment Response

A binary logistic regression analysis was performed to explore the baseline factors associated with achieving a ≥50% reduction in MMD/MHD at month 6. Variables showing an association with treatment response at *p* < 0.10 in univariate analysis were subsequently entered into the multivariable logistic regression model.

In univariate analysis, several baseline variables showed an association with achieving a ≥50% response at month 6. Chronic migraine, compared with HFEM, was associated with lower odds of achieving a ≥50% response, although the association did not reach conventional statistical significance (OR = 0.48, 95% CI, 0.21–1.09; *p* = 0.078).

A higher number of previous preventive treatment failures was significantly associated with lower odds of achieving a ≥50% response (OR = 0.68, 95% CI, 0.48–0.96; *p* = 0.029). Similarly, higher baseline MIDAS score (OR = 0.98, 95% CI, 0.96–0.99; *p* = 0.042) and higher baseline MMD/MHD (OR = 0.96, 95% CI, 0.93–0.99; *p* = 0.044) were associated with lower odds of treatment response.

Medication overuse showed a trend toward a negative association with response (OR = 0.47, 95% CI, 0.21–1.05; *p* = 0.066), although this association did not reach statistical significance. Age (*p* = 0.425), sex (*p* = 0.312), disease duration (*p* = 0.188), presence of aura (*p* = 0.567), BMI (*p* = 0.234), and baseline NRS score (*p* = 0.341) were not significantly associated with achieving a ≥50% response at month 6. Variables meeting the predefined *p* < 0.10 threshold were therefore considered for inclusion in the multivariable model.

Among the 113 patients included in the analytical cohort, 78 (69.0%) achieved a ≥50% reduction in MMD/MHD at month 6 and were classified as responders, while 35 (31.0%) did not meet the predefined response criterion and were classified as non-responders. The five variables identified in the univariate analysis at *p* < 0.10 were entered into the binary logistic regression model. With 78 responders considered as outcome events, the events-per-variable ratio was 15.6. The Hosmer–Lemeshow goodness-of-fit test was non-significant (χ^2^ = 6.74, *p* = 0.565), indicating no evidence of significant lack of fit between the observed and model-predicted outcomes.

The results of the multivariable logistic regression analysis are presented in Table 5.

In the multivariable model, the number of previous preventive treatment failures was the only variable that reached conventional statistical significance. Each additional previous preventive treatment failure was associated with approximately 27% lower odds of achieving a ≥50% response at month 6 (OR = 0.73, 95% CI, 0.54–0.98; *p* = 0.039).

Chronic migraine, baseline MIDAS score, and baseline MMD/MHD retained negative point estimates in the multivariable model but did not reach statistical significance. Medication overuse was also not independently associated with treatment response in the adjusted analysis.

The association between a greater number of previous preventive treatment failures and lower odds of achieving a ≥50% response suggests that greater previous treatment burden may be associated with a less favorable response to anti-CGRP monoclonal antibody therapy. This finding is consistent with observations from other real-world cohorts, including the GARLIT study [16].

The multivariable analysis was exploratory. Although the final model included five predictor variables and 78 outcome events, corresponding to an events-per-variable ratio of 15.6, the sample size and number of candidate predictors should be considered when interpreting the precision and generalizability of the estimates. Overall, the regression analysis identified the number of previous preventive treatment failures as the only statistically significant baseline factor associated with a ≥50% response at month 6, while several other variables showed non-significant trends that warrant evaluation in larger prospective cohorts.

Overall, the findings presented in Section 3 describe the clinical outcomes observed during follow-up in this retrospective real-world cohort. Because the study was observational and did not include a concurrent untreated or active comparator group, the observed changes should be interpreted as associations observed during treatment rather than as evidence of causal treatment effectiveness.

## 4. Discussion

To our knowledge, this multicenter retrospective observational study represents one of the first real-world evaluations of anti-CGRP monoclonal antibody therapy conducted across specialized headache-care centers in Romania. The findings indicate clinically meaningful improvements in headache frequency, acute medication use, pain intensity, and patient-reported measures of headache-related impact and migraine-related disability in both HFEM and CM. The overall pattern of improvement, together with the observed responder rates and predominantly non-serious adverse-event profile, supports the clinical relevance of anti-CGRP therapy in routine headache practice. Because the study was retrospective and lacked a concurrent control group, these findings should be interpreted as observational associations rather than as evidence of a causal treatment effect.

### 4.1. Primary Outcome: Substantial Reduction in Migraine/Headache Frequency

The primary outcome analysis demonstrated a substantial reduction in migraine/headache frequency in both HFEM and CM. In the HFEM group, median MMDs decreased from 10 days at the baseline to 2 days at month 6, while in the CM group, median MHDs decreased from 25 to 6 days over the same period. The corresponding mean reductions were 7.2 ± 3.8 days in HFEM and 16.2 ± 7.4 days in CM. These changes were statistically significant and represent reductions in clinically relevant magnitude across both migraine phenotypes [1,2].

The observed improvement is particularly relevant in the context of the long-standing disease burden of the study population. Mean migraine disease duration was 15.3 ± 9.7 years, indicating that many patients had experienced migraine for a prolonged period before the initiation of anti-CGRP monoclonal antibody therapy. The magnitude of improvement observed despite this prolonged disease history and substantial previous treatment exposure highlights the clinical relevance of headache-frequency reduction in this treatment-experienced population [1,2].

An important feature of the observed response was its early onset. Significant reductions in headache frequency were already evident during the first month of follow-up and were subsequently maintained at the 6- and 12-month assessments. This early and sustained pattern of improvement is consistent with the clinical effects reported for anti-CGRP monoclonal antibodies and may be particularly relevant for patients with frequent or chronic migraine, in whom a reduction in headache burden may rapidly decrease the number of days affected by headache and the need for acute medication [4].

The magnitude of the reduction observed in the present cohort was numerically greater than that reported in several pivotal randomized controlled trials, although the available studies differed in patient populations, treatment conditions, follow-up duration, and timing of outcome assessment. In the REGAIN trial, galcanezumab was associated with a reduction of approximately 4.8 headache days at month 3 in patients with CM, while the CONQUER study reported a reduction of 6.0 MHDs at month 3 [9,10]. Other pivotal trials reported reductions of 3.7 days in episodic migraine with erenumab, 6.6 days in chronic migraine with erenumab, 4.3 days in chronic migraine with fremanezumab, and 4.6 days in episodic migraine with fremanezumab [5,6,11,12]. The PROMISE-2 study of eptinezumab reported a reduction of 8.2 headache days in patients with CM at month 3 [13].

Several methodological and clinical factors may account for the numerical differences between the present cohort and pivotal trials. Real-world populations may differ from randomized trial populations in baseline disease characteristics, previous preventive treatment exposure, comorbidities, and patterns of clinical follow-up. In addition, the principal efficacy comparison in the present study was performed at month 6, whereas several pivotal trials reported their primary efficacy outcomes at month 3. The present cohort also included four anti-CGRP monoclonal antibodies, with treatment selection determined by routine clinical practice; consequently, the unequal distribution of the four agents did not permit meaningful molecule-specific comparative effectiveness analyses.

Taken together, the reduction in headache frequency observed in both HFEM and CM, together with its early onset and persistence during follow-up, indicates a substantial improvement in headache burden in this Romanian real-world cohort. These findings complement the existing evidence from randomized trials by demonstrating clinically relevant headache-frequency reductions in a treatment-experienced population managed under routine clinical conditions.

### 4.2. Secondary Outcomes: Comprehensive Clinical Improvement

The secondary outcomes provide an important complement to the reduction in headache frequency by indicating improvement in acute medication use, pain intensity, headache-related impact, and migraine-related disability. Together, these measures indicate that changes observed during follow-up extended beyond headache frequency alone and included acute medication use, pain intensity, headache-related impact, and migraine-related disability. Reduced reliance on acute medication is particularly relevant in patients at risk of medication overuse, while improvement in patient-reported impact and disability indicates a broader reduction in migraine-related burden.

The interpretation of these patient-reported outcomes should remain instrument-specific. HIT-6 reflects headache-related impact, whereas MIDAS assesses migraine-related disability. The observed changes therefore support improvement in disease burden but should not be interpreted as a direct measure of generic quality of life.

The observed reduction in acute medication use is clinically relevant because medication overuse is associated with increased migraine burden and may contribute to migraine chronification [3]. However, the present study did not directly assess whether the observed reduction in medication use translated into a reduction in medication-related complications. The association between medication overuse and migraine chronification has been well-documented [3].

The improvement in pain severity, as measured by the NRS, by 2–3 points exceeds the minimal clinically important difference of ≥2 points for pain intensity. The substantial reductions in HIT-6 scores (12–15 points) and MIDAS scores (22–50 points) further support improvement in headache-related impact and migraine-related disability. A reduction of ≥8 points in HIT-6 is considered the minimal clinically important difference for this instrument, and our patients far exceeded this threshold. The improvement from Grade IV (severe disability) to Grade II (mild disability) in the HFEM group, and from Grade IV to Grade III in the CM group, represents a meaningful change in the patients’ migraine-related disability.

### 4.3. Responder Rates: High and Sustained Response

A substantial proportion of patients in both migraine subgroups met the predefined ≥50% response criterion during follow-up. The proportion of responders remained relatively stable between the prespecified assessment time points; however, these longitudinal observations should be interpreted cautiously because the study was observational and lacked an untreated or active comparator.

The observed responder rates were broadly comparable with those reported in other real-world cohorts. In the GARLIT study, ≥50% responder rates at month 6 were 76.5% in HFEM and 63.5% in CM [14]. The EARLY study reported an overall ≥50% responder rate of 74.6% at 6 months [15]. The similarity of these estimates provides contextual support for the consistency of clinically meaningful responses observed across different real-world populations receiving anti-CGRP monoclonal antibody therapy.

However, these cross-study comparisons should not be interpreted as evidence that the present treatment strategy was more effective than those evaluated in previous cohorts. Differences in baseline disease burden, previous preventive treatment exposure, treatment selection, migraine phenotype distribution, follow-up duration, and outcome assessment may substantially influence responder rates. In addition, the present study included four different anti-CGRP monoclonal antibodies, whereas individual real-world studies often evaluated a specific molecule. Therefore, the present dataset does not allow for molecule-specific conclusions regarding comparative effectiveness.

The ≥50% responder rate was numerically higher in HFEM than in CM. This difference is compatible with the possibility that patients with episodic migraine may achieve higher response rates than patients with chronic migraine; however, the present study was not specifically designed to establish migraine phenotype as an independent determinant of treatment response. The greater clinical complexity and disease burden typically associated with CM may contribute to differences in treatment response, but such mechanisms cannot be established from the present observational data [3].

More stringent response thresholds further demonstrated substantial treatment responses in a subset of patients. The ≥75% responder analysis indicates that a substantial subset of patients experienced a marked reduction in headache frequency. Although this threshold was less frequently achieved than the conventional ≥50% response, it suggests that the observed benefit was substantial in a clinically relevant subset of patients.

A 100% response, corresponding to zero migraine/headache days at the respective assessment point, was observed in 10.5% of HFEM patients and 6.7% of CM patients at month 6, and in 13.2% and 8.0% at month 12, respectively. Although this represented a minority of patients, the persistence of the outcome at month 12 indicates that a subset of patients achieved a complete absence of recorded migraine/headache days at the respective follow-up assessment.

We deliberately refer to this outcome as a 100% response or zero headache days at the assessment point, rather than as complete remission. The latter term implies a broader and potentially more sustained clinical state that cannot be established solely from the absence of headache days at individual assessment time points.

The high proportion of patients achieving ≥75%, and in a smaller subgroup, 100% reduction represents an important finding because these stringent responder thresholds provide information beyond the conventional ≥50% response criterion. Nevertheless, the interpretation of these outcomes should take into account the retrospective design, the absence of a control group, and the possibility of differences in follow-up completeness and patient selection.

Previous real-world studies of erenumab have also reported clinically meaningful response rates, including patients achieving responses above 30% and 50%, with a smaller proportion reaching complete response [15]. The higher responder proportions observed for some endpoints in the present cohort may reflect differences in patient selection, baseline disease characteristics, treatment exposure, or duration of follow-up. Because these factors were not directly compared across studies, they should be regarded as potential explanations rather than demonstrated causes.

Taken together, the responder analyses indicate that a substantial proportion of patients in both the HFEM and CM groups achieved clinically meaningful reductions in headache frequency, with responses maintained over the 12-month observation period. The presence of ≥75% and 100% responders further demonstrates that a subset of patients experienced particularly large reductions in headache burden, although these findings require confirmation in larger prospective cohorts.

### 4.4. Conversion from Chronic to Episodic Migraine and Medication Overuse Discontinuation

The conversion from chronic to episodic migraine represented an important clinical outcome in the present cohort. Among the 75 patients who fulfilled the criteria for chronic migraine at the baseline, 53 patients (70.7%) no longer met the frequency criterion for chronic migraine at month 6, having fewer than 15 headache days per month. This proportion remained relatively stable at month 12, when 52 patients (69.3%) fulfilled the conversion criterion.

The observed conversion indicates that a substantial proportion of patients with chronic migraine experienced a reduction in headache frequency sufficient to move below the diagnostic frequency threshold for chronic migraine during treatment. Given the greater disease burden generally associated with chronic compared with episodic migraine, this reduction in headache frequency may have important clinical relevance [3]. However, conversion based on headache-day frequency alone should not be interpreted as demonstrating a complete reversal of the underlying migraine disorder or as evidence of improved long-term prognosis.

The relatively stable conversion rate between month 6 and month 12, decreasing only slightly from 70.7% to 69.3%, indicates that the reduction in headache frequency was maintained in a substantial proportion of the cohort during longer-term follow-up. Nevertheless, because the present study was retrospective and lacked an untreated or active comparator group, these findings should be interpreted as an association with treatment exposure rather than definitive evidence of a causal “dechronification” effect.

The conversion rate at month 6 was numerically lower than that reported in the GARLIT study (70.7% vs. 77.2%) [15]. Several differences between the cohorts may have contributed to this variation. The prevalence of medication overuse was 76.0% in the present CM cohort compared with 85.3% in GARLIT; however, the available data do not allow us to determine whether this difference influenced the observed conversion rates. Other differences in baseline disease characteristics, treatment exposure, patient selection, and follow-up procedures may also have contributed.

The 1-year follow-up data from GARLIT identified factors associated with persistent treatment response, including triptan response, lower BMI, and a ≥50% reduction in MMDs during the first month. These findings provide useful context for the potential heterogeneity of long-term response in real-world populations; however, they should not be extrapolated directly to the present cohort because the predictive factors identified in GARLIT were not specifically evaluated as predictors of persistent response in the current study.

Medication overuse discontinuation represented another clinically relevant outcome. Among the 67 patients with medication overuse at the baseline, 51 patients (76.1%) no longer fulfilled the predefined criteria for medication overuse at month 6, while 50 patients (74.6%) remained below the medication-overuse threshold at month 12. The reduction in medication overuse occurred in parallel with the substantial decrease in headache frequency and monthly analgesic-use days observed during treatment.

Medication overuse is an important component of the clinical burden of migraine and is associated with greater headache frequency and disability [3]. The high proportion of patients no longer fulfilling medication-overuse criteria during follow-up therefore represents an important clinical finding. However, the present study cannot establish whether anti-CGRP therapy directly interrupted the pathophysiological cycle of medication overuse.

The observed reduction in medication overuse is biologically plausible in the context of the substantial reduction in headache frequency and pain intensity observed in the cohort. Reduced headache frequency may decrease the need for acute medication, while improved pain control may further reduce the frequency of acute medication use. These mechanisms should, however, be regarded as plausible explanations rather than mechanisms demonstrated by the present study, because medication-taking behavior and treatment expectations were not prospectively assessed.

Overall, the findings suggest that anti-CGRP monoclonal antibody treatment was associated with both a substantial reduction in headache frequency sufficient to meet the criterion for conversion from CM to episodic migraine in a large proportion of patients and a marked reduction in medication overuse among patients affected at the baseline.

### 4.5. Treatment Discontinuation and Persistence

During the observed follow-up period, 22 out of 113 patients (19.5%) discontinued anti-CGRP monoclonal antibody treatment. Lack of efficacy was the most frequently documented reason for discontinuation (12 patients, 10.6%), followed by adverse events (3 patients, 2.7%), pregnancy or planned pregnancy (2 patients, 1.8%), patient decision unrelated to efficacy (3 patients, 2.7%), and loss to follow-up (2 patients, 1.8%).

The overall discontinuation rate indicates that the majority of patients remained on treatment during the observed follow-up period. The proportion discontinuing because of adverse events was relatively small (2.7%), although the presence of treatment discontinuations attributable to adverse events indicates that tolerability was not uniformly optimal for all patients.

The observed discontinuation rate was lower than rates frequently reported for some oral migraine preventive therapies, in which adverse effects and insufficient efficacy are important contributors to treatment discontinuation [3]. However, direct comparison of persistence between treatment classes should be interpreted cautiously because persistence estimates are strongly influenced by treatment duration, study design, reimbursement policies, follow-up intensity, definitions of discontinuation, and patient selection.

Kaplan–Meier analysis showed estimated treatment-persistence probabilities of 86.5% at 6 months, 82.3% at 12 months, and 75.2% at 18 months. These estimates indicate that a substantial proportion of patients remained on anti-CGRP monoclonal antibody treatment during the observed follow-up period. Because treatment persistence was analyzed using a time-to-discontinuation approach, patients who remained on treatment at the end of their available follow-up contributed as censored observations. The persistence estimates should therefore be interpreted in the context of the retrospective study design, the available follow-up information, and the clinical criteria used to define treatment discontinuation.

A real-world study evaluating fremanezumab reported treatment continuation rates of 68.3%, 58.0%, and 50.6% at 12, 18, and 24 months, respectively. The persistence estimates observed in the present cohort were numerically higher at the corresponding follow-up points. However, such comparisons should be interpreted descriptively because differences in patient populations, treatment exposure, follow-up duration, discontinuation definitions, and healthcare systems may substantially affect treatment persistence.

The relatively high treatment persistence observed in the present cohort may be compatible with sustained clinical benefit and acceptable tolerability. However, the present study did not directly measure patient motivation, treatment satisfaction, or the individual contribution of efficacy and tolerability to treatment continuation. Therefore, these factors should be regarded as potential explanations rather than established determinants of persistence.

The findings nevertheless highlight the importance of evaluating treatment persistence alongside efficacy and safety outcomes in real-world migraine populations. For a chronic disorder requiring long-term preventive management, sustained treatment exposure is clinically relevant, particularly when it is accompanied by persistent reductions in headache frequency, acute medication use, and disability.

### 4.6. Safety Profile: Favorable and Consistent with Clinical Trials

The safety findings indicate a generally favorable observed tolerability profile, with adverse events predominantly non-serious and treatment discontinuation because of adverse events uncommon. This profile is clinically reassuring for long-term preventive therapy, although the retrospective design and reliance on routine clinical documentation may limit the detection and characterization of uncommon or delayed adverse events.

No serious adverse events were documented during the study period. However, three patients (2.7%) discontinued treatment because of adverse events, indicating that although treatment discontinuation attributable to adverse events was uncommon, tolerability was not optimal in all patients. Accordingly, the present findings support a generally favorable observed tolerability profile but should not be interpreted as evidence that adverse events did not influence treatment persistence.

The overall adverse-event rate in the present cohort (24.8%) was higher than the 10.3% reported in the GARLIT study [14]. This difference may reflect several methodological and clinical factors, including differences in patient populations, treatment exposure, follow-up procedures, adverse-event ascertainment, and the inclusion of multiple anti-CGRP monoclonal antibodies in the present cohort. Because adverse events were assessed retrospectively, differences in documentation practices may also have contributed to the observed variation. However, the available data did not allow us to determine which of these factors was responsible for the difference.

The frequency of local injection-site reactions in the present study (7.1%) was within the range reported in clinical trials of anti-CGRP monoclonal antibodies [9,10]. The corresponding rate reported in GARLIT was lower (approximately 2%) [14]. This difference should be interpreted cautiously because differences in adverse-event ascertainment and reporting procedures may influence the observed frequencies across studies. It should not be interpreted as evidence that the GARLIT study systematically underestimated injection-site reactions.

The occurrence of less frequently reported events, including alopecia (3.5%) and menstrual irregularities (2.7%), is noteworthy in the context of real-world safety monitoring. Such events may be detected more readily in routine clinical practice because of broader patient populations and longer observation periods. However, the present study was not designed to establish a causal relationship between these events and anti-CGRP monoclonal antibody exposure, and their relatively low frequency limits interpretation.

The SAFE-CGRP study, a multicenter retrospective evaluation of CGRP pathway-targeting monoclonal antibodies in patients with relevant comorbidities or clinical conditions that may have limited representation in randomized trials, reported no serious adverse events during extended follow-up and provided additional real-world safety data in clinically complex populations. The absence of documented serious adverse events in the present cohort is consistent with these observations.

Overall, the present findings indicate that adverse events were reported in approximately one-quarter of the cohort, were predominantly non-serious, and resulted in treatment discontinuation in a small proportion of patients (2.7%). These findings provide supportive real-world evidence regarding the tolerability of anti-CGRP monoclonal antibodies while recognizing that the retrospective design and relatively limited sample size restrict the ability to detect uncommon or delayed adverse events.

### 4.7. Predictive Factors of Treatment Response

The multivariable analysis identified the number of previous preventive treatment failures as the only statistically significant independent predictor of achieving a ≥50% response at month 6. This finding suggests that greater previous treatment burden may be associated with a lower probability of response, although the observational design does not establish causality and previous treatment failure may also represent a marker of greater disease complexity.

This association suggests that a greater history of previous preventive treatment failure may identify patients with a lower probability of achieving a ≥50% response to anti-CGRP monoclonal antibody therapy. The finding is clinically relevant because patients referred for anti-CGRP therapy after multiple preventive failures often represent a population with a greater cumulative treatment burden. However, the present analysis could not determine whether previous treatment failure itself directly reduces the probability of response or instead serves as a marker of greater disease complexity.

The findings are broadly consistent with observations from other real-world cohorts. The GARLIT study identified a lower number of previous preventive treatment failures as being associated with a greater effectiveness of galcanezumab in patients with CM [14]. In addition, the 1-year GARLIT follow-up study identified triptan response, lower BMI, and a ≥50% reduction in MMDs during the first month as factors associated with persistent response. Similarly, a Korean real-world study evaluating CGRP monoclonal antibodies in elderly patients identified previous failure of onabotulinumtoxinA as a negative predictor of treatment response [26].

The consistency of these observations across independent real-world cohorts suggests that previous treatment history may be clinically informative when estimating the probability of response. Nevertheless, the specific predictors identified across studies are not uniform, emphasizing the heterogeneity of treatment response and the influence of differences in patient populations, treatment exposure, outcome definitions, and analytical methods.

In the present study, chronic migraine, higher baseline MIDAS score, medication overuse, and higher baseline MMD/MHD showed negative point estimates in the multivariable model, but none reached conventional statistical significance. These findings may indicate a possible association between greater baseline disease burden and lower probability of response; however, the confidence intervals and *p*-values do not provide sufficient evidence to consider these variables independent predictors in the present cohort.

This interpretation is broadly compatible with findings from other predictive studies. A recently developed nomogram for response to anti-CGRP monoclonal antibodies identified several clinical characteristics associated with treatment response, including migraine phenotype, age, baseline headache frequency, medication overuse, and acute medication use [27]. The variability of predictors across real-world studies highlights the complexity of predicting individual treatment response and the need for validation in larger, prospectively characterized cohorts.

From a clinical perspective, the association between multiple previous preventive treatment failures and lower odds of response should not be interpreted as a reason to withhold anti-CGRP monoclonal antibody therapy from treatment-experienced patients. Even within the present cohort, patients with previous preventive failures demonstrated clinically meaningful improvements in headache frequency and other outcome measures. Rather, the finding may help clinicians establish realistic expectations regarding the probability of response in patients with a greater previous treatment burden.

The potential clinical value of earlier intervention is also supported by real-world evidence suggesting that baseline disease burden and headache frequency may influence treatment response [26,27]. However, the present study was not designed to determine whether earlier initiation of anti-CGRP therapy results in superior long-term outcomes. Prospective studies specifically evaluating treatment timing, cumulative preventive treatment exposure, and response trajectories will therefore be required before definitive recommendations regarding earlier intervention can be made.

Similarly, treatment strategies such as combination preventive therapy may represent potential approaches for patients with incomplete response, but these strategies were not evaluated in the present study and should therefore be regarded as areas for future investigation rather than conclusions derived from the current dataset.

Overall, the regression analysis suggests that previous preventive treatment burden is associated with treatment response, while the effects of other clinical characteristics remain uncertain. These findings support the need for larger prospective studies incorporating detailed clinical, behavioral, and treatment-history variables to develop and externally validate clinically useful prediction models for anti-CGRP treatment response.

### 4.8. Comparison with the Italian GARLIT Study

The Italian GARLIT study provides a useful real-world reference for interpreting the findings of the present Romanian cohort because both studies evaluated anti-CGRP monoclonal antibody treatment in routine clinical practice [15]. At month 6, the ≥50% responder rates were broadly similar between the two cohorts: 78.9% versus 76.5% in HFEM and 64.0% versus 63.5% in CM in the present study and GARLIT, respectively.

The similarity of these estimates provides contextual evidence that substantial treatment responses can be observed in different European real-world headache populations. However, these findings should not be interpreted as demonstrating equivalence between the two cohorts or generalizability across healthcare systems, because the studies differed in treatment exposure, patient characteristics, healthcare organization, and methodological procedures.

Several baseline differences should be considered when interpreting the observed outcomes. The present cohort was younger on average (42.6 vs. 47.1 years), included a higher proportion of women (95.6% vs. 80.5%), and had a higher proportion of HFEM patients (33.6% vs. 20.2%) than the GARLIT cohort. Medication overuse was also less frequent in the overall present cohort (59.3%) than the proportion reported for GARLIT (85.3%).

Baseline headache frequency also differed between the cohorts. Importantly, among the patients with CM, the present cohort had higher, rather than lower, median baseline MHDs (25 vs. 21 days). Therefore, the difference in CM-to-episodic conversion rates cannot be attributed simply to higher baseline MHDs in GARLIT. The CM-to-episodic conversion rate was 70.7% in the present cohort compared with 77.2% in GARLIT, while medication-overuse discontinuation was 76.1% and 82.0%, respectively [15]. Differences in baseline characteristics, treatment exposure, and follow-up methodology may have contributed to these variations, but their relative contribution cannot be established from the available data.

The overall adverse-event rate was higher in the present cohort than in GARLIT (24.8% vs. 10.3%). Several factors may account for this difference, including differences in adverse-event ascertainment and documentation, patient characteristics, treatment exposure, and the inclusion of multiple anti-CGRP monoclonal antibodies in the present cohort. Because the two studies used different real-world settings and methodological approaches, the difference should not be interpreted as evidence of a clinically meaningful difference in safety between the cohorts.

In particular, the lower adverse-event frequency reported in GARLIT should not be interpreted as evidence of underreporting without direct comparative information regarding adverse-event ascertainment. Overall, the similarities in responder rates, together with the broadly comparable clinical outcomes, provide useful contextual support for the effectiveness of anti-CGRP monoclonal antibody therapy in routine European headache practice [15].

### 4.9. Comparison with Other Real-World Studies

The findings of the present study were also considered in the context of other real-world investigations of anti-CGRP monoclonal antibody therapy.

The EARLY study, which evaluated erenumab in 170 Italian patients, reported a ≥50% responder rate of 74.6% at 6 months [14]. The PEARL study, conducted across 11 European countries and including 1140 patients treated with fremanezumab, reported that 58.5% of patients achieved a ≥50% reduction in MMDs during the 6-month follow-up period [19]. The FINESSE study, which followed 1016 patients in Germany and Austria for up to 24 months, reported ≥50% responder rates of 57.0% in episodic migraine and 47.8% in chronic migraine [28]. In the TRIUMPH Europe study, which compared galcanezumab with traditional oral preventive treatments in 717 patients from Germany, Italy, Spain, and the United Kingdom, the 3-month weighted ≥50% responder rate was 63.1% overall, including 65.0% in CM and 60.7% in episodic migraine [29].

Real-world evidence is also available for eptinezumab. A prospective UK analysis reported that after one infusion, 51% of patients achieved at least a 30% reduction, 35% achieved a ≥50% reduction, and 13% achieved a ≥75% reduction in MMDs [30]. A French real-world study involving patients with difficult-to-treat migraine reported ≥50% responder rates of 35.4% at month 3, 39.0% at month 6, and 45.7% at month 12 [31].

The findings of this multicenter retrospective observational study have several potential implications for migraine management in Romania. The observed improvement across headache frequency, acute medication use, pain intensity, patient-reported headache impact, and migraine-related disability supports the clinical relevance of anti-CGRP monoclonal antibody therapy in appropriately selected patients managed in routine practice. These findings may complement, rather than replace, evidence from randomized trials and international real-world studies.

The populations differed substantially in baseline disease severity, previous treatment exposure, migraine phenotype, treatment selection, follow-up duration, and outcome definitions. In particular, some of the eptinezumab cohorts included highly treatment-refractory populations, which may partially explain their lower observed responder rates [30,31].

The variation between real-world studies illustrates the substantial heterogeneity of routine clinical migraine populations. Treatment response may be influenced by baseline headache frequency, previous preventive treatment failures, medication overuse, comorbidities, treatment selection, and duration of follow-up. Consequently, cross-study comparisons are most appropriately interpreted as contextual rather than comparative effectiveness analyses.

Taken together, the available real-world evidence demonstrates that clinically meaningful responses to anti-CGRP monoclonal antibodies can be observed across different European healthcare settings and patient populations. The present Romanian cohort contributes additional evidence from a population that has been relatively underrepresented in published real-world studies. Nevertheless, the heterogeneity between studies emphasizes the need for prospective multicenter studies using standardized outcome definitions and comparable follow-up schedules.

### 4.10. Clinical Implications

The findings of this multicenter retrospective observational study have several potential implications for migraine management in Romania.

First, the observed reductions in headache frequency and the substantial ≥50% responder rates in both HFEM and CM indicate that anti-CGRP monoclonal antibody therapy is associated with clinically meaningful improvement in this real-world Romanian cohort. The responder rates observed at month 6 were broadly comparable with those reported in several European real-world studies [15,19,28,29,30,31,32]. These findings provide local clinical evidence that may complement randomized trial data and existing international real-world evidence.

Second, the 70.7% conversion rate from CM to episodic migraine at month 6 and the 76.1% rate of discontinuation of medication overuse among patients affected at the baseline indicate that substantial reductions in headache frequency and acute medication use can occur during treatment. These outcomes are clinically relevant because chronic migraine and medication overuse are associated with greater disease burden and disability [3]. However, these findings should be interpreted as associations observed during treatment and should not be taken as proof that anti-CGRP therapy reverses the underlying process of migraine chronification or directly interrupts medication-overuse mechanisms.

Third, the observed safety and treatment-persistence data support the feasibility of maintaining anti-CGRP monoclonal antibody therapy over extended follow-up in routine practice. Adverse events were reported in 24.8% of patients, no serious adverse events were documented, and 19.5% discontinued treatment during the observed follow-up period. Nevertheless, adverse events accounted for treatment discontinuation in 2.7% of patients, indicating that tolerability should continue to be assessed systematically during long-term treatment.

The present findings provide real-world observations regarding the clinical outcomes associated with anti-CGRP monoclonal antibody treatment in patients with HFEM and CM. They may complement existing evidence from randomized trials and other real-world studies, but they should not be interpreted as establishing comparative or causal treatment effectiveness. However, the statement that these therapies should be considered first-line preventive treatment cannot be directly derived from this study, because treatment sequencing was determined by clinical practice and the study did not compare anti-CGRP monoclonal antibodies with conventional preventive treatments. Their precise position in the treatment algorithm should therefore remain dependent on current clinical guidelines, regulatory indications, reimbursement criteria, and individual patient characteristics.

Fourth, the identification of the number of previous preventive treatment failures as the only statistically significant independent predictor of ≥50% response provides potentially useful information for clinical decision-making. Each additional previous preventive treatment failure was associated with approximately 27% lower odds of achieving a ≥50% response. This finding may help clinicians identify patients with a greater previous treatment burden who may require more individualized expectations and closer follow-up. Importantly, however, the association should not be interpreted as a reason to withhold anti-CGRP therapy from treatment-experienced patients.

The finding also raises the possibility that treatment timing may influence outcomes, as patients with extensive previous preventive treatment exposure appeared less likely to achieve a ≥50% response. Nevertheless, the present study did not directly compare early versus delayed initiation of anti-CGRP therapy and therefore cannot establish that earlier treatment improves outcomes. Prospective studies specifically designed to evaluate treatment timing are needed before such an implication can be translated into treatment recommendations [16,17].

Finally, the consistency of the present findings with several European real-world cohorts provides useful contextual evidence for clinicians practicing in Romania and other settings where local real-world data remain limited [15,29,31,32]. Nevertheless, the observed similarities should not be interpreted as proof that treatment effectiveness is identical across healthcare systems or patient populations. Rather, the findings support the clinical relevance of anti-CGRP monoclonal antibody therapy in routine Romanian headache practice and provide a basis for future prospective, multicenter studies involving larger and more systematically characterized populations.

### 4.11. Strengths and Limitations

#### 4.11.1. Strengths

The present study has several strengths that contribute to the growing real-world evidence regarding anti-CGRP monoclonal antibody therapy in migraine.

First, to our knowledge, this is the first multicenter real-world study from Romania to evaluate clinical outcomes associated with anti-CGRP monoclonal antibody therapy across patients with HFEM and CM. The study therefore provides locally generated clinical data from a Romanian headache-care setting, an area in which published real-world evidence remains limited. This is particularly relevant in the context of differences in healthcare organization, treatment access, reimbursement frameworks, and patient characteristics between Eastern and Western European countries.

Second, the study included four anti-CGRP monoclonal antibodies—galcanezumab, erenumab, fremanezumab, and eptinezumab—within the same overall real-world cohort. This allowed the study to capture treatment outcomes across a broader spectrum of anti-CGRP therapies than studies restricted to a single molecule. However, because treatment groups were markedly unequal in size, the present study should not be considered as a comparative effectiveness study between individual monoclonal antibodies. The principal strength of including multiple agents is therefore the broader representation of routine clinical anti-CGRP treatment rather than the ability to determine the superiority of one agent over another.

Third, the study incorporated longitudinal follow-up extending to 12 months, with treatment-persistence information available beyond the primary 12-month efficacy assessment in a subset of patients. This extended observation period allowed for the evaluation of changes in clinical outcomes over time, as well as treatment discontinuation, medication-overuse status, and adverse events. Longitudinal real-world data are particularly relevant for migraine prevention because treatment decisions involve sustained management rather than short-term efficacy alone [14,15].

Fourth, the study evaluated a broad range of clinically relevant outcomes, including migraine/headache frequency, acute medication use, pain intensity, HIT-6, MIDAS, responder thresholds, conversion from chronic to episodic migraine, medication-overuse discontinuation, treatment persistence, and adverse events. The simultaneous assessment of these outcomes provides a multidimensional characterization of changes in migraine burden rather than relying exclusively on headache frequency.

Fifth, the retrospective review included the documentation of a range of adverse events, including less frequently reported events such as alopecia, menstrual irregularities, and injection-site reactions. Such real-world safety information may complement evidence generated in randomized controlled trials, where patient eligibility criteria, follow-up duration, and adverse-event ascertainment may differ from routine clinical practice [7,8,9,10,11,12,13].

Sixth, the study included patients with both HFEM and CM, including individuals with medication overuse and substantial previous preventive treatment exposure. This reflects the clinical heterogeneity encountered in specialized headache practice and provides information regarding treatment outcomes in patients who may differ from the more selectively defined populations enrolled in randomized clinical trials [7,8,9,10,11,12,13].

Finally, the study incorporated a multivariable exploratory analysis of predictors of treatment response. The identification of previous preventive treatment failures as an independent factor associated with lower odds of achieving a ≥50% response provides a clinically relevant hypothesis for future prospective studies. Although this finding requires external validation, the analysis adds an exploratory predictive dimension beyond the descriptive assessment of treatment effectiveness.

From a mechanistic perspective, the clinical benefits observed with anti-CGRP monoclonal antibodies should be interpreted within the multifactorial pathophysiology of migraine. CGRP plays a central role in trigeminovascular activation, neurogenic inflammation, and peripheral and central sensitization, providing a strong biological rationale for CGRP-targeted therapies [33]. However, migraine involves a broader network of interacting neural, inflammatory, immune, and metabolic mechanisms. Emerging evidence also supports a role for the gut–brain axis, with bidirectional interactions that may influence migraine frequency, severity, and disability through pathways that extend beyond systemic inflammatory signaling [34]. These observations emphasize that the clinical effects of anti-CGRP monoclonal antibodies should be considered within a complex biological framework in which CGRP-mediated mechanisms represent an important, but not isolated, component of migraine pathophysiology.

#### 4.11.2. Strengths and Limitations

The present study has several strengths, including its multicenter real-world design in Romania, inclusion of patients with both high-frequency episodic migraine and chronic migraine, evaluation of four anti-CGRP monoclonal antibodies, longitudinal assessment of clinical outcomes through 12 months, and the inclusion of clinically relevant measures such as headache frequency, acute medication use, disability, treatment persistence, and adverse events. The study also provides exploratory information on factors associated with treatment response and contributes real-world data from a Romanian specialized headache-care setting, where published evidence remains limited. However, the findings should be interpreted in light of several limitations. The retrospective observational design is subject to selection and information bias and residual confounding, and the absence of a concurrent comparator prevents the causal attribution of observed changes to anti-CGRP treatment. The sample size was limited for subgroup and molecule-specific analyses, particularly because galcanezumab accounted for 89 out of 113 patients (78.8%), while the numbers treated with erenumab, fremanezumab, and eptinezumab were small. The cohort was also predominantly female and was recruited from two specialized headache centers, which may limit generalizability to other clinical settings and to male patients. In addition, retrospective data collection resulted in variability in documentation, and the use of last observation carried forward for longitudinal analyses may introduce bias in the presence of informative missingness or treatment discontinuation. The open-label clinical setting may also have influenced the patient-reported outcomes, while the absence of detailed information on adherence, treatment expectations, comorbidities, lifestyle factors, and other potential confounders may have resulted in residual confounding. Finally, the relatively limited sample size and follow-up constrain the ability to detect uncommon or delayed adverse events. Accordingly, the present findings should be considered as real-world observational evidence of clinical outcomes rather than definitive evidence of causal or comparative effectiveness.

### 4.12. Future Research Directions

Future research should focus on larger, prospective, multicenter studies with longer follow-up to validate the present findings, improve the completeness and standardization of longitudinal data collection, and better characterize the long-term effectiveness, safety, and treatment persistence of anti-CGRP monoclonal antibodies in Romanian and Eastern European populations. Adequately powered comparative studies with more balanced treatment groups are also needed to evaluate potential differences between individual anti-CGRP monoclonal antibodies while accounting for baseline disease characteristics, previous preventive treatment exposure, and other relevant confounders.

Further studies should also investigate predictors of treatment response, treatment continuation and discontinuation, and outcomes in clinically relevant subgroups that may be underrepresented in randomized trials. Incorporating patient-centered outcomes, healthcare utilization, quality of life, and health-economic measures, together with standardized prospective data collection across multiple centers, may help support individualized treatment selection and optimize the long-term management of migraine with anti-CGRP monoclonal antibodies.

## 5. Conclusions

This multicenter retrospective observational study provides real-world evidence on the clinical outcomes of anti-CGRP monoclonal antibody treatment in Romanian patients with high-frequency episodic migraine and chronic migraine. During follow-up, substantial reductions in migraine/headache frequency, acute medication use, pain intensity, and migraine-related disability were observed. At month 6, ≥50% responder rates were 78.9% in HFEM and 64.0% in CM, while 70.7% of patients with CM met the predefined criterion for conversion to episodic migraine and 76.1% of patients with baseline medication overuse no longer fulfilled the predefined criteria for medication overuse. Treatment persistence at 12 months was 82.3%, adverse events were predominantly non-serious, and no serious adverse events were documented. Overall, the findings indicate clinically relevant improvements across multiple dimensions of migraine burden during treatment in this Romanian real-world cohort.

## Figures and Tables

**Table 1:** Baseline demographic and clinical characteristics of the study population.

Characteristic	Total (*N* = 113)	HFEM (*n* = 38)	CM (*n* = 75)	*p*-Value
Age (years), mean ± SD	42.6 ± 10.8	40.5 ± 9.6	43.7 ± 11.2	0.124
Female sex, *n* (%)	108 (95.6%)	36 (94.7%)	72 (96.0%)	0.762
Disease duration (years), mean ± SD	15.3 ± 9.7	12.8 ± 8.4	16.6 ± 10.1	0.041 *
Aura present, *n* (%)	17 (15.0%)	5 (13.2%)	12 (16.0%)	0.695
Medication overuse, *n* (%)	67 (59.3%)	10 (26.3%)	57 (76.0%)	<0.001 *
BMI (kg/m^2^), mean ± SD	24.2 ± 4.1	23.5 ± 3.8	24.6 ± 4.3	0.183
Previous preventives failed, mean ± SD	2.8 ± 1.4	2.4 ± 1.2	3.0 ± 1.5	0.026 *
Baseline MMD/MHD, median (IQR)	18 (12–28)	10 (8–12)	25 (18–30)	<0.001 *
Baseline MPI, median (IQR)	15 (8–20)	10 (6–14)	20 (15–25)	<0.001 *
Baseline NRS, median (IQR)	8 (7–9)	7 (6–8)	8 (7–9)	0.038 *
Baseline HIT-6, median (IQR)	68 (62–72)	64 (60–68)	70 (66–72)	<0.001 *
Baseline MIDAS, median (IQR)	56 (30–72)	30 (25–50)	70 (56–80)	<0.001 *

Abbreviations: BMI, body mass index; CM, chronic migraine; HFEM, high-frequency episodic migraine; IQR, interquartile range; MHD, monthly headache days; MMD, monthly migraine days; MPI, monthly painkiller intake; NRS, Numerical Rating Scale; HIT-6, Headache Impact Test; MIDAS, Migraine Disability Assessment Scale; SD, standard deviation. * *p* < 0.05.

**Table 2:** Changes in secondary outcome variables at month 6.

Outcome	HFEM Group (*n* = 38)	CM Group (*n* = 75)
MPI, median change from baseline	−7 (IQR, −10 to −4) *	−14 (IQR, −18 to −9) *
NRS, median change from baseline	−2 (IQR, −3 to −1) *	−3 (IQR, −4 to −2) *
HIT-6, median change from baseline	−12 (IQR, −16 to −8) *	−15 (IQR, −20 to −10) *
MIDAS, median change from baseline	−22 (IQR, −35 to −12) *	−50 (IQR, −60 to −35) *

Abbreviations: CM, chronic migraine; HFEM, high-frequency episodic migraine; IQR, interquartile range; MIDAS, Migraine Disability Assessment Scale; MPI, monthly painkiller intake; NRS, Numerical Rating Scale; HIT-6, Headache Impact Test. * *p* < 0.001 for comparison with baseline.

**Table 3:** Responder rates at months 1, 3, 6, and 12.

Response Threshold	Time Point	HFEM, *n*/*N* (%)	95% CI	CM, *n*/*N* (%)	95% CI
≥50%	Month 1	24/38 (63.2%)	47.3–77.0	40/75 (53.3%)	42.0–64.2
≥50%	Month 3	27/38 (71.1%)	55.0–83.5	47/75 (62.7%)	51.3–72.8
≥50%	Month 6	30/38 (78.9%)	63.5–89.2	48/75 (64.0%)	52.6–74.1
≥50%	Month 12	28/38 (73.7%)	57.9–85.3	45/75 (60.0%)	48.5–70.5
≥75%	Month 6	13/38 (34.2%)	20.6–50.7	25/75 (33.3%)	23.5–44.6
≥75%	Month 12	12/38 (31.6%)	18.6–47.9	22/75 (29.3%)	20.0–40.6
100%	Month 6	4/38 (10.5%)	3.7–24.6	5/75 (6.7%)	2.6–15.0
100%	Month 12	5/38 (13.2%)	5.2–27.7	6/75 (8.0%)	3.3–16.5

Abbreviations: CM, chronic migraine; CI, confidence interval; HFEM, high-frequency episodic migraine. Note: The number of patients available for responder analysis was 113 at each assessment time point (38 with HFEM and 75 with CM).

**Table 4:** Adverse events reported during the study period.

Adverse Event	Total (*N* = 113)	HFEM (*n* = 38)	CM (*n* = 75)
Any adverse event, *n* (%)	28 (24.8%)	8 (21.1%)	20 (26.7%)
Local injection-site reaction	8 (7.1%)	2 (5.3%)	6 (8.0%)
Constipation	6 (5.3%)	2 (5.3%)	4 (5.3%)
Hair loss/alopecia	4 (3.5%)	1 (2.6%)	3 (4.0%)
Menstrual irregularities	3 (2.7%)	1 (2.6%)	2 (2.7%)
Fatigue	2 (1.8%)	0 (0%)	2 (2.7%)
Dizziness	2 (1.8%)	1 (2.6%)	1 (1.3%)
Dysphonia	1 (0.9%)	0 (0%)	1 (1.3%)
Weight gain	1 (0.9%)	0 (0%)	1 (1.3%)
Pseudogripal syndrome/flu-like symptoms	1 (0.9%)	1 (2.6%)	0 (0%)

Abbreviations: CM, chronic migraine; HFEM, high-frequency episodic migraine. Percentages for individual adverse events were calculated using the total number of patients in each respective group as the denominator.

**Table 5:** Multivariable logistic regression analysis of baseline predictors of ≥50% response at month 6.

Variable	B	S.E.	Wald	*p*-Value	Odds Ratio	95% CI
Chronic migraine	−0.82	0.48	2.91	0.088	0.44	0.17–1.13
Number of previous preventive treatment failures	−0.31	0.15	4.27	0.039	0.73	0.54–0.98
Baseline MIDAS	−0.02	0.01	3.14	0.076	0.98	0.96–1.00
Medication overuse	−0.59	0.48	1.51	0.219	0.55	0.21–1.42
Baseline MMD/MHD	−0.05	0.03	3.65	0.056	0.95	0.90–1.00

Abbreviations: B, regression coefficient; CI, confidence interval; MIDAS, Migraine Disability Assessment Scale; MMD/MHD, monthly migraine/headache days; S.E., standard error *p* < 0.05. Outcome: ≥50% response at month 6. Responders: *n* = 78; non-responders: *n* = 35. Five predictor variables were included in the final multivariable model; events-per-variable ratio = 15.6.

## Data Availability

The data presented in this study are available on request from the corresponding author. The data are not publicly available due to privacy and ethical restrictions.
